# An Increase in Tobacco Craving Is Associated with Enhanced Medial Prefrontal Cortex Network Coupling

**DOI:** 10.1371/journal.pone.0088228

**Published:** 2014-02-05

**Authors:** Amy C. Janes, Stacey Farmer, Blaise deB. Frederick, Lisa D. Nickerson, Scott E. Lukas

**Affiliations:** McLean Imaging Center, McLean Hospital, Harvard Medical School, Belmont, Massachusetts, United States of America; Wake Forest School of Medicine, United States of America

## Abstract

Craving is a key aspect of drug dependence that is thought to motivate continued drug use. Numerous brain regions have been associated with craving, suggesting that craving is mediated by a distributed brain network. Whether an increase in subjective craving is associated with enhanced interactions among brain regions was evaluated using resting state functional magnetic imaging (fMRI) in nicotine dependent participants. We focused on craving-related changes in the orbital and medial prefrontal cortex (OMPFC) network, which also included the subgenual anterior cingulate cortex (sgACC) extending into the ventral striatum. Brain regions in the OMPFC network are not only implicated in addiction and reward, but, due to their rich anatomic interconnections, may serve as the site of integration across craving-related brain regions. Subjective craving and resting state fMRI were evaluated twice with an ∼1 hour delay between the scans. Cigarette craving was significantly increased at the end, relative to the beginning of the scan session. Enhanced craving was associated with heightened coupling between the OMPFC network and other cortical, limbic, striatal, and visceromotor brain regions that are both anatomically interconnected with the OMPFC, and have been implicated in addiction and craving. This is the first demonstration confirming that an increase in craving is associated with enhanced brain region interactions, which may play a role in the experience of craving.

## Introduction

Craving, or the strong desire to use an abused substance, is a key component of drug addiction, and is a motivator for drug use [1], [2]. To understand craving, research has focused on identifying the neurobiological substrates underlying the desire to use drugs. However, craving is linked with a number of brain regions, such as those involved in primary drug reward, habitual drug use, memory, and reward based-decision making [3]–[8]. Thus, it is hypothesized that a distributed brain network mediates craving instead of a single localized region [9]. An increase in craving may, therefore, involve enhanced brain region interactions that facilitate integration of information across these disparate brain areas.

The relationship between craving and brain-network interactions can be evaluated using functional magnetic resonance imaging (fMRI) data collected at rest. While previous task-related fMRI research has identified discrete brain regions associated with craving [6], [8], resting-state fMRI allows for the evaluation of brain function at a more distributed network level. During rest, brain regions with highly correlated fluctuations in blood oxygen level dependent (BOLD) signals are defined as functional networks [10], called resting state networks (RSNs), which are thought to reflect intrinsic functional brain organization [11], [12]. Subsequent research has revealed that RSNs are associated with known brain systems related to cognition, perception, and reward [13], [14]. To evaluate associations between craving and network-level brain changes, we collected resting-state fMRI and subjective craving data in nicotine-dependent smokers at two time points approximately one hour apart. Over this one-hour time period, participants reported a significant rise in craving as measured by the brief questionnaire of smoking urges (QSU) [15].

We focused on the orbital and medial prefrontal cortex (OMPFC) network, which is a previously defined RSN [13], [16] comprised of anatomically interconnected brain regions [17] involved in the mesocorticolimbic reward circuit. Specifically, the OMPFC network contains the medial and orbital prefrontal cortex (mPFC, OFC), and the subgenual anterior cingulate cortex (sgACC) extending into the ventral striatum. We focused on this network as these prefrontal and striatal regions are implicated in craving [4], [7], [18] drug reinforcement [19], [20], and reward processing [21]. Additionally, the OFC is thought to be a multimodal integration area leading to hedonic experience [22]. In addition to the direct link between OMPFC brain regions and craving, this network may act as a hub [23] where information is integrated to facilitate the subjective experience of craving. To confirm the idea that a distributed brain network is associated with craving, we hypothesize that interactions between the OMPFC network and other craving-related brain regions will increase along with a rise in the subjective experience of craving.

## Methods

### Participants

Seventeen nicotine-dependent smokers (8 men/9 women) were studied: 25.4±4.6 (mean ± standard deviation) years old with 15.3±2.1 years of education and 6.7±4.7 pack-years of smoking use (pack years  =  number of packs of cigarettes smoked/day x years as a smoker). Participants had an average Fagerström test for nicotine dependence (FTND) [24] score of 6.3±1.0, which confirmed moderate to severe nicotine dependence. Participants also reported smoking ≥10 cigarettes/day over the past 6 months. The Structured Clinical Interview for DSM-IV (SCID) was used to assess all participants for current nicotine dependence and to exclude those with a lifetime diagnosis of the following conditions: organic mental disorder, bipolar or unipolar depression, or schizophrenia spectrum disorder. Participants also were excluded for pregnancy, current psychotropic drug use or recent alcohol use (Alco-Sensor FST, Intoximeters, Inc.). No participants consumed any alcohol prior to the study as indicated by a blood alcohol level of 0. Additionally, no subjects met criteria for alcohol abuse or dependence. Recruitment was conducted using online advertisements and fliers posted in the Boston area. All participants gave written informed consent prior to participating in the study and the institutional review board at McLean Hospital approved this study and consent procedure.

### Functional Neuroimaging

All participants smoked one of their own cigarettes immediately following signing the informed consent to standardize the time since a cigarette was last smoked. MRI scanning began approximately 1.5 h after smoking this cigarette. Scans were acquired on a Siemens Trio 3 Tesla scanner (Erlangen, Germany) with a 32-channel head coil. Multiecho multiplanar rapidly acquired gradient-echo (ME-MPRAGE) structural images were acquired with the following parameters (TR  =  2.1 s, TE 3.3 ms, slices  =  128, matrix  =  256×256, flip angle  =  7°, resolution  =  1.0 mm×1.0 mm×1.33 mm), and gradient echo echo-planar images were acquired using the following parameters (TR  =  2.5 s, TE  =  30 ms, flip angle  =  90°, slices  =  42, voxel size  =  3.5 mm isotropic). Slices were acquired aligned to the anterior and posterior commissure and the phase encode direction was set to acquire from the posterior to anterior direction to prevent prefrontal signal loss. During the 6-minute resting state fMRI scans participants were asked to remain awake with their eyes open. Two resting state scans were acquired approximately 1 h apart.

### fMRI Pre-processing

All data analysis was conducted using tools from the Functional Magnetic Resonance Imaging of the Brain (FMRIB) Software Library (FSL; www.fmrib.ox.ac.uk/fsl). Functional data pre-processing included: motion correction with MCFLIRT, brain extraction using BET, slice timing correction, spatial smoothing with a Gaussian kernel of full-width half-maximum 6 mm, and high-pass temporal filter with Gaussian-weighted least-squares straight-line fitting with σ = 100 s. Subject specific data was registered to the MNI152 2 mm^3^ standard space template (Montreal Neurological Institute, Montreal, QC, Canada) using FLIRT and the fMRI data was transformed into standard space at 2×2×2 mm resolution using the registration transformation matrices.

### fMRI Resting-State Independent Components Analysis

**Figure 1:**
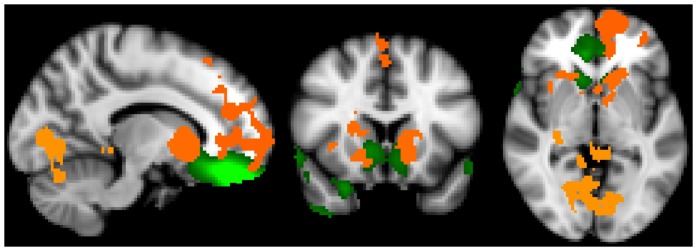
Left to right: Sagittal, coronal, and axial views of grouped analyses demonstrating that craving increases along with orbital and medial prefrontal cortex (OMPFC) network coupling. Green voxel highlighting is the OMPFC network defined by the group ICA. The orange overlay represents voxels with increased coupling to the OMPFC network as craving increases.

To identify resting state networks common to all participants, the data from all subjects were temporally concatenated and a multivariate group probabilistic ICA (PICA) was conducted using FSL MELODIC [25], [26]. Consistent with our prior work [16], the dimensionality was fixed to 35 to investigate large-scale RSNs. To ensure stable convergence of the ICA, the ICA was run 8 times followed by a meta-level ICA fed by all of the spatial maps from the 8 decompositions [14]. This meta ICA was conducted to identify the set of independent components common to all subjects. These components included, common resting state networks, which have been identified elsewhere [13], [14] such as the default mode, salience, fronto-parietal, motor, and visual networks. Artifact-related components were also identified. Through comparison with brain networks reported previously [13], [14], [16] the resulting independent component maps were visually inspected to identify the OMPFC network (see Fig. 1, green shading).

### Dual Regression

To calculate subject specific time courses and spatial maps, we used a dual regression approach [27]–[29]. In the first stage of dual regression, the full set of group ICs, which include all RSNs and artifact components, are used in a multiple spatial regression against each individual subject's dataset to estimate the average time course of voxels in each RSN. By including all of the ICs in the multiple regression, any voxel with contributions from multiple signal sources (for example, from coupling with an RSN and from motion effects) will have these effects partialled out into their separate contributions by the multiple regression prior to the averaging. The subject-specific time courses were normalized to unit variance and then used in a second multiple regression against the individual subject's dataset, to identify voxels correlated with each of the RSN time courses, thus identifying the spatial map of each RSN unique to the subject. To evaluate a change in functional connectivity between the two resting state acquisitions, difference maps were calculated by subtracting the individual subject specific spatial maps for the second minus the first resting state session.

### Resting State Associations with Craving and Carbon Monoxide

Just prior to the first resting state scan and just after the second resting state scan, craving was measured by the brief 10-item Questionnaire of Smoking urges [15]. Expired carbon monoxide (CO; Micro Smokerlyzer II, Bedfont Scientific Instruments) was also measured at these two time periods. Significant differences in craving and CO at these two time periods were assessed using a paired t-test. Changes in craving and CO were calculated by subtracting the second (post-scan) minus the first (pre-scan) measurements. Changes in craving and CO were correlated with the RSN difference maps using non-parametric permutation testing using 5,000 permutations (FSL Randomize) [30]. Multiple comparisons were corrected to p<0.05 using cluster-based thresholding where the cluster-forming threshold was Z = 2.3 [31].

## Results

### Craving and Carbon monoxide

Craving, measured by the QSU, significantly increased following the second resting state scan (t_16_ = −3.3, *p*<0.01; pre 22.5±8.2, post 30.2±10.2). Expired CO levels significantly dropped from pre (26.9±12.3 ppm) to post (18.6±8 ppm) scanning (t_16_ = 6.3, *p*<0.01). There was no relationship between the change in craving and CO, nor was the difference in craving or CO associated with age, pack-year, or FTND score.

### Identified resting state network

The OMPFC network common to all subjects included the ventromedial PFC (Broadmann area (BA) 10), orbitofrontal cortex (OFC; BA 11), subgenual ACC (BA 24, 32), and the ventral striatum/nucleus accumbens extending into the adjacent caudate. (Figure 1, green shading). This definition of the OMPFC network visually overlapped with our previous work [16] and the work of others [13].

### Craving associations with RSNs

**Figure 2:**
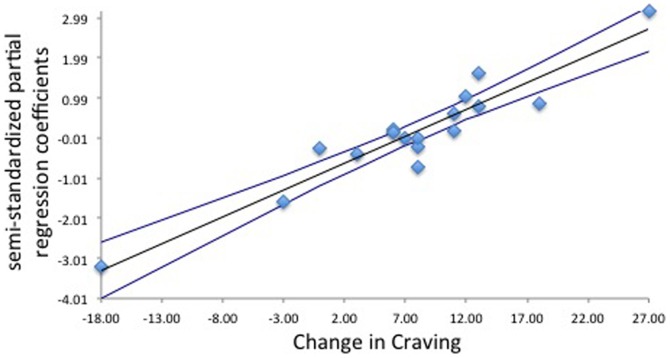
Difference in craving (post-scan minus pre-scan) associated with semi-standardized partial regression coefficients. Coefficients were extracted from the subject specific spatial maps from stage 2 of the dual regression for regions that showed statistically significant relationships with craving in the whole brain analysis (as such, this plot is only meant to supplement our inference and may overestimate the true effect size).

No associations were found between the first assessment of craving and the first RSN measure, nor were associations found between the second craving assessment and the second RSN measure. A positive correlation was found between the change in craving (second – first) and the OMPFC RSN difference maps (second – first). As craving rose, increased connectivity was found between the OMPFC network difference map and several brain regions including: left dorsal regions of the superior frontal gyrus (BA 10) extending into the dACC (BA 24, 32) and left frontal pole (BA 10), bilateral supplementary motor area (BA 6), bilateral ventral striatum, bilateral caudate, bilateral ventral occipital cortex (BA 18, 19), right thalamus, right hippocampus and parahippicampal gyrus, and left superior cerebellum (left crus 1 and left lobule VI; Figure 1, orange shading; related correlation plot, Figure 2; Table 1).

**Table 1:** Brain regions showing enhanced network coupling with the OMPFC network as craving increases.

Brain Area	Brodmann Area	X	Y	Z	P-Max	Voxels
Bilateral parahippocampal gyrus, bilateral occipital cortex, left cerebellum, right hippocampus	18, 19	−38	−62	−36	0.02	2507
Bilateral anterior ventral striatum (nucleus accumbens extending into the caudate), right mid to posterior caudate		12	22	−20	0.03	2242
Supplementary motor area, left frontal pole, superior frontal gyrus, dorsal anterior cingulate cortex	6, 10, 24, 32	−10	54	−18	0.03	2190

Brain areas and Brodmann area refer to the location of each cluster of contiguous voxels. MNI coordinates (X, Y, Z) refer to the region of maximum coupling for each cluster. P-max refers to the maximum P-statistic in each cluster (p_cluster corrected_ <0.05). Voxels refers to the total number of voxels within the cluster.

### Carbon Monoxide associations with RSNs

No associations were found between CO and any of the RSN measurements.

## Discussion

The current results identify that a relationship exists between increased subjective craving and enhanced coupling between the OMPFC network and several other brain regions. This association was noted only when evaluating the *change* in craving as no interaction was found between craving and RSN measures at either time point one or two alone. This suggests that stable levels of craving are not related to network functional connectivity strength, but experiencing a rise in craving is accompanied by greater OMPFC network coupling. While others have evaluated the relationship between nicotine withdrawal symptoms and the default mode and executive control networks [28], this is the first focused evaluation of craving on reward-related brain networks. As we found no correlation between the decrease in expired CO and OMPFC network coupling, the relationship between craving and changes in connectivity cannot be attributed to physiological changes due to a drop in CO.

There is a strong link between the OMPFC network and craving as regions within this network, which include the vmPFC, OFC, sgACC, and striatum, activate during craving [4], [32]. Several, but not all studies also report deactivation of these regions during the regulation of craving [4], [32]–[35]. Supporting the idea that OMPFC regions act as a craving-related functional unit, Hanlon and colleagues [4] showed that this entire cluster of brain regions activates during tobacco craving. More broadly the OMPFC network may be involved in reward evaluation during the experience of craving, as OMPFC network regions are active during both reward anticipation and reward delivery [36]–[40].

In the present study, increased subjective craving paralleled enhanced coupling between the OMPFC network and brain regions that typically activate when drug users are exposed to drug-associated cues; including dorsal regions of the mPFC [41], the hippocampus [42], visual areas [41], [43], sensory motor regions [44], the striatum [7], [45], and cerebellum [46]. This co-activation of prefrontal and other cortical, limbic, striatal, and visceromotor areas led London and colleagues [18] to speculate that there is an “interplay of related networks” during drug-cue exposure that may correspond with craving. Our findings confirm that a rise in subjective craving is correlated with enhanced coupling between brain regions associated with craving, which are also anatomically connected with the OMPFC [17]. These rich interconnections with OMPFC brain regions are thought to form a sensory-visceromotor link to guide reward-related behavior and give rise to hedonic experience [17], [22]. Thus, it is possible that the integration of information between these regions may play a role in the experience of craving as well as guiding smoking behavior.

In the context of the current study, we are unable to directly determine whether there is a link between increased brain network coupling and behavior. However, as craving intensity peaks prior to relapse [47], it is tempting to speculate that that the enhanced coupling between brain regions associated with increased craving facilitates smoking-related behavior. Increased craving-related coupling between the OMPFC network and regions such as the supplementary motor area (SMA) supports the notion that the observed network-interactions may have an impact on behavior. Not only does the SMA activate to smoking-related cues [44], but SMA neurons fire prior to hand movements, which is thought to facilitate psychomotor responses to an object [48]. Thus, the interaction between these brain regions implicated in craving and behavior may actually be involved in the process of “preparing to smoke”. Alternatively, as smokers were in the scanner and unable to smoke *ad libitum*, when they had the immediate desire, the involvement of brain regions such as the SMA may regulate smoking behavior as the SMA also is implicated in the inhibition of action [49].

Future directions not only include linking network changes with behavior, but also defining the neurotransmitter systems mediating brain network connectivity. For instance, greater midbrain dopamine D3 receptor availability is positively correlated with enhanced coupling between the OMPFC network and brain structures such as the striatum and OFC [50]. In addition, D3 receptors play a strong role in nicotine-seeking and are a promising target for nicotine cessation treatment [51]–[53]. These studies suggest that future research should focus on the role of D3 receptors in craving-related OMPFC network coupling. Given that D3 receptors influence other addictive disorders [54], these future studies should be expanded to include craving-related network changes for nicotine and other abused substances. While we found no relationship between craving and factors such as age and level of nicotine dependence, these factors should also be studied more directly as our work focused specifically on relatively young and heavily nicotine dependent smokers. Finally, while we speculate that OMPFC brain regions may act as an integrative hub [23], future studies involving alternative connectivity methods should focus on identifying the directionality of information flow between craving-related brain structures.

Our results confirm that increased tobacco craving is associated with enhanced interactions between reward- and craving-related brain regions. This rise in craving over time was not related to baseline measures of smoking history, nor was the change in brain interactions due to a decrease in expired CO, indicating that changes in OMPFC coupling are specifically related to enhanced subjective craving. The relationship between changes in craving and RSN connectivity indicate that brain network interactions are associated with changes in subjective state.
